# Metagenomic Assembly and Draft Genome Sequence of an Uncharacterized *Prevotella* sp. from Nelore Rumen

**DOI:** 10.1128/genomeA.00723-15

**Published:** 2015-07-09

**Authors:** Luciano T. Kishi, Raphael B. de Jesus, Claudio D. Pavani, Eliana G. M. Lemos, Jackson A. M. de Souza

**Affiliations:** a UNESP, Univ Estadual Paulista, Departamento de Tecnologia/LBMP, Jaboticabal, SP, Brazil; b UNESP, Univ Estadual Paulista, Departamento de Biologia/LGA, Jaboticabal, SP, Brazil

## Abstract

*Prevotella* is one of the most abundant genera in bovine rumen, although no genome has yet been assembled by a metagenomics approach applied to Brazilian Nelore. We report the draft genome sequence of *Prevotella* sp., comprising 2,971,040 bp, obtained using the Illumina sequencing platform. This genome includes 127 contigs and presents a low 48% GC.

## GENOME ANNOUNCEMENT

*Prevotella* species have been isolated from a number of environments, including the human oral cavity (1), human skin abscesses (2), and soils (3). *Prevotella* species are also prevalent within the rumen and gastrointestinal tracts of herbivores and omnivores. The bacterial isolates used to establish the ruminal *Prevotella* group were originally described by Bryant et al. (4) on the basis of the nutritional requirement for hemin and were initially divided into two subspecies, *Bacteroides ruminicola* subsp. *ruminicola* (type strain 23) and *B. ruminicola* subsp. *brevis* (type strain GA33).

Various ruminal bacteria, including *Prevotella ruminicola* 23, have been shown to have esterase activity (5). However, knowledge regarding the diversity of esterases present in the genome, including the regulation, biochemical characteristics, and mechanistic action of these enzymes, is limited. Recently, the complete genome sequence of *P. ruminicola* 23 was made available (6), therefore providing an opportunity to evaluate the repertoire of enzymes enabling this organism to function as a highly efficient hemicellulose-degrading bacterium.

We collected ruminal content from fistulated 24-month-old Nelore cattle. Total DNA was extracted using the FastDNA SPIN kit (MP Biomedical, LLC). DNA sequencing was performed using the Illumina HiscanSQ platform. *De novo* assembly was carried out using IDBA_UD (7). Binning of the assembled contigs was carried out based on metagenomic read coverage, tetranucleotide frequency, and the occurrence of unique marker genes using MaxBin (8), which generated 127 clustered contigs. Our search of the 127 contigs against all bacterial and archaeal genome sequences available in the GenBank database (January 2015) using BLASTn (9) showed best hits for *P. ruminicola* 23. The sequence composition-based binning, the sequence homology search, and the metagenomic read recruitment collectively indicated that the 127 contigs represent well the draft genome sequence of the single-species population.

The draft genome is 2,971,040 bp, with 48% GC content (*N*_50_ value, 109,266), and contains a total of 2,515 protein-coding genes, 1 rRNA, and 48 tRNAs. Gene prediction and functional annotation of the draft genome were performed using the RAST server (10). Further analysis of carbohydrate-active enzymes using the dbCAN database (11) have indicated the presence of 39 glycoside hydrolases, 43 glycosil transferases, 4 polysaccharide lyases, 20 carbohydrate esterases, 6 auxiliary activities, and 25 carbohydrate-binding modules.

Functional genomics research is rapidly emerging, and its application via a metagenomics approach has revealed an important tool for the discovery and development of new and alternative pathways for biotechnology purposes. Our advances in understanding the biology and ecology of ruminal *Prevotellaceae* will significantly improve future applications for ruminant growth, nutrition, and even plant biomass conversion technologies.

### Nucleotide sequence accession number. 

The sequences were deposited in the NCBI with accession number SRX818104.
